# Rapid clearance of epigenetic protein reporters from wound edge cells in *Drosophila* larvae does not depend on the JNK or PDGFR/VEGFR signaling pathways

**DOI:** 10.1002/reg2.12

**Published:** 2014-06-23

**Authors:** Aimee E. Anderson, Michael J. Galko

**Affiliations:** ^1^ Department of Biochemistry and Molecular BiologyHoustonTX77030USA; ^2^ Genes and Development Graduate ProgramHoustonTX77030USA; ^3^ University of Texas MD Anderson Cancer CenterUnit 1000, 1515 Holcombe BoulevardHoustonTX77030USA

**Keywords:** *Drosophila*, epigenetic, polycomb, trithorax, wound healing

## Abstract

The drastic cellular changes required for epidermal cells to dedifferentiate and become motile during wound closure are accompanied by changes in gene transcription, suggesting corresponding alterations in chromatin. However, the epigenetic changes that underlie wound‐induced transcriptional programs remain poorly understood partly because a comprehensive study of epigenetic factor expression during wound healing has not been practical. To determine which chromatin modifying factors might contribute to wound healing, we screened publicly available fluorescently tagged reporter lines in *Drosophila* for altered expression at the wound periphery during healing. Thirteen reporters tagging seven different proteins showed strongly diminished expression at the wound edge. Three downregulated proteins, Osa, Kismet, and Spt6, are generally associated with active chromatin, while four others, Sin3A, Sap130, Mi‐2, and Mip120, are associated with repressed chromatin. In all cases reporter downregulation was independent of the Jun N‐terminal kinase and Pvr pathways, suggesting that novel signals control reporter clearance. Taken together, our results suggest that clearance of chromatin modifying factors may enable wound edge cells to rapidly and comprehensively change their transcriptional state following tissue damage.

## Introduction

When skin is injured, rapid and efficient repair of the wound is essential to restore the barrier, prevent infection and blood loss, and restore the function of the epidermis. During healing, cells at the wound edge must act in concert to quickly seal the gap (Shaw and Martin 2009b; Razzell et al. 2011). These cells must cease their normal differentiated activities and become motile, migrating in a coordinated manner toward the gap and changing their adhesive properties to cover and seal the wound. At the molecular level, these activities are accomplished, at least in part, by profound changes in gene expression. In *Drosophila*, transcriptional changes in response to wounding include induction of *misshapen* (*msn*), *puckered* (*puc*) (Galko and Krasnow 2004; Lesch et al. 2010), *Dopa decarboxylase* (*Ddc*), *pale* (*ple*) (Mace et al. 2005), *stitcher* (*stit*) (Wang et al. 2009), *krotzkopf verkehrt* (*kkv*) (Pearson et al. 2009), *Flotillin‐2* (*Flo‐2*), *Src oncogene at 42A* (*Src42A*) (Juarez et al. 2011), *chickadee* (*chic*) (Brock et al. 2012) and myriad other genes (Patterson et al. 2013). In the epidermis, transcriptional changes probably include both the repression of many genes required for the cell's differentiated function and activation of genes required for motility, such as those regulating the actin cytoskeleton. Previous work has shown that key signaling pathways, including the Jun N‐terminal kinase (JNK) (Ramet et al. 2002; Javelaud et al. 2003; Li et al. 2003; Galko and Krasnow 2004; Bosch et al. 2005; Campos et al. 2010; Lesch et al. 2010) and platelet derived growth factor receptor/vascular endothelial growth factor receptor (PDGFR/VEGFR; Pvr in *Drosophila*) pathways (Lynch et al. 1987; Bao et al. 2009; Wu et al. 2009), are critical regulators of wound healing. Downstream, important transcription factors include Grainy head (grh) (Mace et al. 2005), Jun (Campos et al. 2010), and Fos (Lesch et al. 2010). However, how the presumably complex downstream transcriptional outputs of these pathways are coordinated during the rapid events of wound healing remains poorly understood.

Chromatin modification is a critical mechanism for regulating gene expression in many biological processes, such as cell cycle regulation, development, and differentiation (reviewed by Goldberg et al. 2007; Kouzarides 2007). These mechanisms can include changes in nucleosome spacing, composition, and covalent modifications to histones, including methylation, acetylation, phosphorylation, and ubiquitination. Chromatin can be modified by a vast number of highly conserved proteins, which generally act in multiprotein complexes to define transcriptionally active or repressed regions. Hence, changes in the expression of chromatin modifying proteins can affect the expression of multiple downstream genes in parallel. This suggests that chromatin modifying proteins may play a role in processes that require the rapid, coordinated alteration of gene expression, such as wound healing. Indeed, epigenetic regulation has been implicated in many regenerative processes (Marumo et al. 2008; Stewart et al. 2009; Jopling et al. 2010; Palacios et al. 2010; Katsuyama and Paro 2011; Taylor and Beck 2012; Tseng and Levin 2012; reviewed by Palacios and Puri 2006; Guasconi and Puri 2009; Slattery et al. 2009; Yakushiji et al. 2009; Barrero and Izpisua Belmonte 2011).

Many repressively acting chromatin modifying proteins fall into the polycomb group (PcG) class of genes, while some proteins associated with active genes are categorized in the trithorax group (trxG) (reviewed by Schwartz and Pirrotta 2008). A previous study investigating expression of epigenetically acting factors during wound healing in murine skin observed a downregulation of three repressive PcG proteins, Eed, Ezh2, and Suz12, and increased expression of two activating trxG members, Jmjd3 and Utx (Shaw and Martin 2009a). These results suggested a model in which a global decrease in repressive factors and increase in activating factors at the wound edge is employed by wound edge cells to broadly upregulate expression of target genes. However, a comprehensive survey of the epigenetic contribution to wound healing has not been performed. Screening large numbers of factors for expression changes in mammalian model systems is impractical; therefore, we sought to test the hypothesis that chromatin modifying proteins are differentially expressed during wound healing using our previously established *Drosophila* wound closure model (Galko and Krasnow 2004; Lesch et al. 2010). To accomplish this, we made use of existing GFP‐ and YFP‐tagged protein trap lines (Morin et al. 2001; Kelso et al. 2004; Buszczak et al. 2007; Quinones‐Coello et al. 2007; Ryder et al. 2009). We screened all available lines that trap chromatin modifying proteins for altered expression in the vicinity of healing wounds. Here we describe 13 reporter lines, trapping seven different proteins, whose expressions are decreased at the wound edge. These 13 reporters fall into two distinct temporal patterns during wound healing. Surprisingly, the proteins trapped in the reporter lines encompass both activating and repressive activities, suggesting a complex model of epigenetic regulation during wound healing.

## Results

### A screen for altered expression of epigenetic reporters during wound closure

**Figure 1:**
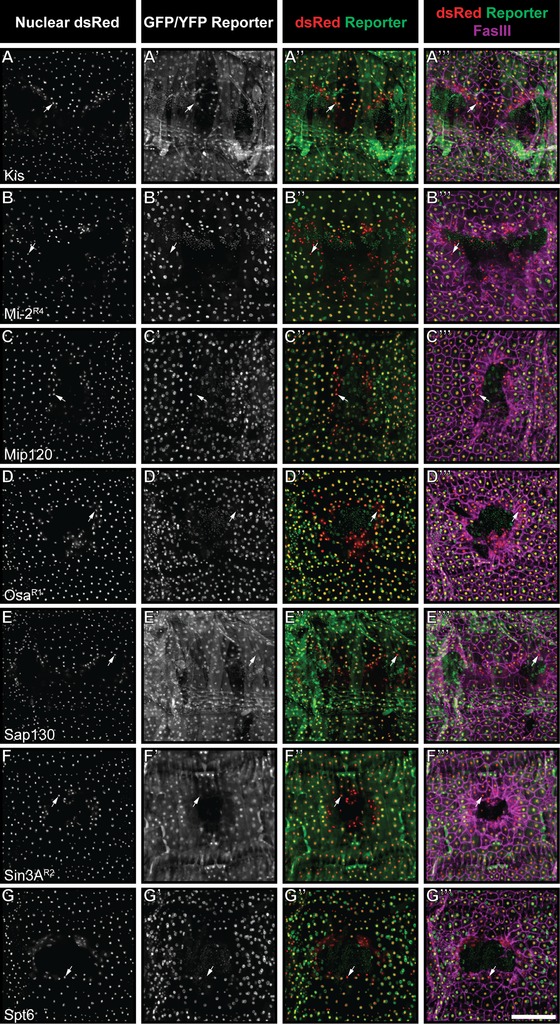
Reporters for seven proteins are downregulated in the vicinity of the wound. (A)−(G′′′) Dissected epidermal whole mounts of larvae heterozygous for *e22c‐Gal4, UAS‐DsRed2‐Nuc* and the indicated reporter transgene were immunostained for Fasciclin III 4 h post‐wounding. For all genotypes, DsRed panels (A)−(G), (A′′)−(G′′), (A′′′)−(G′′′) show the location of epidermal nuclei while reporter panels (A′)−(G′), (A′′)−(G′′), (A′′′)−(G′′′) highlight all cells expressing the reporter. Fasciclin III staining (magenta, A′′′−G′′′) highlights epidermal cell membranes and the wound edge. Arrows indicate epidermal nuclei that have lost detectable reporter expression. Scale bar 200 μm.

We were interested in identifying epigenetic proteins that might regulate wound healing. We made use of existing protein trap resources to identify any reporter corresponding to a protein involved in epigenetic regulation that showed differential expression at the wound edge. We screened 54 independent GFP‐ or YFP‐ tagged protein trap lines from the Flytrap and Cambridge Protein Trap Insertion (CPTI) collections (Morin et al. 2001; Kelso et al. 2004; Buszczak et al. 2007; Quinones‐Coello et al. 2007; Ryder et al. 2009). The selected lines tag known or suspected proteins involved in epigenetic regulation, including proteins that directly interact with histones, proteins found in chromatin modifying complexes, and those that contain critical domains common to known epigenetic factors. Most of the reporters examined were expressed in multiple cell types, including the underlying muscles (for an example see Fig. 1D−D′′′). Therefore, to specifically identify epidermal nuclei, we crossed each reporter to an epidermal *Gal4* (*e22c‐Gal4*) driving *UAS‐DsRed2nuc* (Lesch et al. 2010) and considered only nuclei expressing DsRed when evaluating reporter expression at the wound edge. For the initial screen we examined wounded larvae (Galko and Krasnow 2004; Lesch et al. 2010) 4 h post‐wounding as this is a time‐point when the wound edge cells are actively migrating (Wu et al. 2009) and transcriptional changes have been observed at the wound edge (Galko and Krasnow 2004; Lesch et al. 2010; Baek et al. 2012; Brock et al. 2012; Stevens and Page‐McCaw 2012). In the majority of reporter lines, the GFP or YFP signal in the epidermis was too weak for live microscopy. We therefore visualized reporter expression by immunostaining dissected whole mounts with an anti‐GFP antibody that recognizes both GFP and YFP. To label wound edge membranes, the epidermis was also stained with anti‐Fasciclin III. The majority of the reporters showed no obvious difference between distal and proximal nuclei, and none of the reporters screened showed increased expression at the wound edge (see Table S1 for a list of reporters without wound‐edge‐specific staining patterns, and Fig. S1 for a representative example). However, we observed a striking decrease in the expression of 13 reporters in wound‐proximal cells (see arrows in Figs. 1 and S2). These 13 reporters tag seven different proteins involved in epigenetic regulation: Osa, Sin3A, Sap130, Kismet, Mi‐2, Mip120, and Spt6 (Table 1). Two of the lines tag Osa, two tag Sin3A, and five tag Mi‐2. Interestingly, these reporters corresponded to chromatin remodeling proteins that are thought to be both activators (Osa, Spt6, Kismet) and repressors (Mi‐2, Sin3A, Sap130, and Mip120). Two additional lines with decreased expression were also identified (asterisks in Table S1), but because it is unclear whether they tag Mi‐2 or the overlapping Su(Tpl) protein, these lines were not included in subsequent analyses.

**Table 1:** Reporter lines described in the current study

Line	Source	Designation	Driver	Primary effect on transcription	Epidermal wound RNAi phenotype
*osa* ^*CC00445*^	Flytrap	Osa^R1^	*e22c‐Gal4*	Activating	Lethal
*osa* ^*CPTI003089*^	CPTI/DGRC	Osa^R2^			
*Sin3A* ^*P01869*^	Flytrap	Sin3A^R1^	*A58‐Gal4*	Activating	Closed wounds
*Sin3A* ^*YB0058*^	Flytrap	Sin3A^R2^			
*Mi‐2* ^*CPTI000020*^	CPTI/DGRC	Mi‐2^R1^	*e22c‐Gal4*	Repressing	Closed wounds
*Mi‐2* ^*CPTI001119*^	CPTI/DGRC	Mi‐2^R2^			
*Mi‐2* ^*CPTI000106*^	CPTI/DGRC	Mi‐2^R3^			
*Mi‐2* ^*CA06598*^	Flytrap	Mi‐2^R4^			
*Mi‐2* ^*YD0067*^	Flytrap	Mi‐2^R5^			
*kis* ^*CPTI003576*^	CPTI/DGRC	Kis	*A58‐Gal4*	Activating	Closed wounds
*Mip120* ^*P02006*^	Flytrap	Mip120	*A58‐Gal4*	Repressing	Closed wounds
*Sap130* ^*CPTI001478*^	CPTI/DGRC	Sap130	*e22c‐Gal4*	Repressing	Closed wounds
*Spt6* ^*CA07692*^	Flytrap	Spt6	*e22c‐Gal4*	Repressing	Lethal

Null mutants or strong hypomorphs of the proteins tagged in our screen are lethal at the embryonic or early larval stages (e.g. Kennison and Tamkun 1988; Treisman et al. 1997; Kehle et al. 1998; Spradling et al. 1999; Bourbon et al. 2002; Petruk et al. 2006). Therefore, if these tags do not interfere with protein function, then larvae homozygous for the tags should be viable. We tested this (Table 2): 71% of the proteins identified generated viable late stage larvae when homozygous for the reporter in question. For these, we then tested another function—whether the homozygous larvae could successfully close epidermal wounds. In all cases they could (Table 2). Taken together, these results suggest that the protein tags in the homozygous viable lines do not disrupt protein function. The localization data of the lethal lines, where there is some disruption of protein function, should be interpreted with more caution.

**Table 2:** Homozygous phenotypes of reporter lines

Designation	% Open wounds	Standard deviation	Homozygous viability
Osa^R1^	0%	0%	Viable
Osa^R2^	0%	0%	Viable
Sin3A^R1^	0%	0%	Viable
Sin3A^R2^	0%	0%	Viable
Mi‐2^R1^	Lethal	−	Lethal
Mi‐2^R2^	Lethal	−	Lethal
Mi‐2^R3^	Lethal	−	Rare escapers
Mi‐2^R4^	Lethal	−	Lethal
Mi‐2^R5^	Lethal	−	Lethal
Kis	0%	0%	Viable
Mip120	0%	0%	Viable
Sap130	Lethal	−	Lethal
Spt6	0%	0%	Viable
*w* ^*1118*^	2%	4%	
bsk^RNAi^	100%	0%	

Because the reporters are cleared from wound edge cells we did not anticipate that their knockdown within the epidermis would necessarily lead to a defect in wound closure. Nevertheless, this is simple to test via tissue‐specific RNAi targeting each gene and subsequent evaluation of wound closure (Table 1). Epidermal expression of RNAi transgenes targeting *osa* and *Spt6* under the control of *e22c‐GAL4* was lethal, probably reflecting a developmental or physiological role for these genes in the epidermis and preventing further analysis. However, animals expressing RNAi transgenes targeting *Sin3A*, *Mi‐2*, *kis*, *Mip120*, or *Sap130* were viable and had no obvious morphological or wound closure defects, suggesting that these factors are dispensable for normal cell architecture and healing in the larval epidermis.

**Figure 2:**
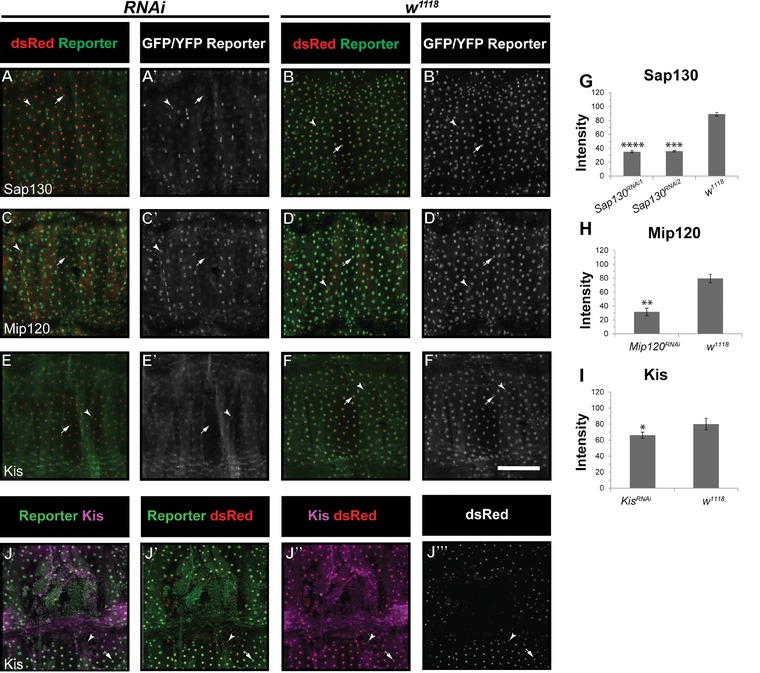
RNAi knockdown reduces expression of the Sap130, Mip120, and Kis reporters. (A)−(F′) Unwounded segments of dissected epidermal whole mounts of larvae heterozygous for an epidermal Gal4 driver (see Table 1), *UAS‐DsRed2nuc*, the indicated reporter, and the indicated *UAS‐RNAi*. Larvae were raised at 30°C. (A)−(A′) Sap130 reporter with *UAS‐Sap130* ^*RNAi1*^. (B)−(B′) Sap130 reporter with *w* ^*1118*^. (C)−(C′) Mip120 reporter with *UAS‐Mip120* ^*RNAi*^. (D)−(D′) Mip120 reporter with *w* ^*1118*^. (E)−(E′) Kis reporter with *UAS‐Kis* ^*RNAi*^. (F)−(F′) Kis reporter with *w* ^*1118*^. Arrowheads, muscle nuclei that do not express dsRed2Nuc; arrows, epidermal nuclei; note the lack of GFP staining in (A)−(A′), (C)−(C′), and (E)−(E′) compared with controls. (G), (I) Quantification of reporter intensity in epidermal nuclei of indicated genotypes. *P < 0.05; **P < 0.005; ***P < 0.001; ****P < 0.0005 (Student's *t* test). For the remaining reporter/RNAi combinations tested no statistically significant reduction in signal was observed. (J)−(J′′′′) Kis antibody staining (J, J′′, J′′′′) is reduced at the wound edge 4 h post‐wounding (J′′), and overlaps with Kis reporter staining (J). Scale bar 200 μm.

Next, we tested if the tagged proteins accurately reflect the expression of the native proteins. We raised larvae bearing a reporter, its corresponding RNAi transgene, and an epidermal Gal4 driver and *UAS‐dsRed2Nuc*, at 29°C to maximize epidermal RNAi expression. We then compared the expression level of each reporter in epidermal nuclei when its cognate gene was targeted (Fig. 2A−I). RNAi lines for Sap130 (Fig. 2A−C), Mip120 (Fig. 2D,F), and Kis (Fig. 2G,I) yielded a statistically significant reduction in reporter signal compared with controls. For the RNAi transgenes targeting the remaining genes, we did not see a statistically significant reduction in antibody signal. In these cases, we cannot distinguish between insufficient knockdown from the RNAi transgenes or inaccurate tagging of unintended proteins. Because of this, the localization and RNAi wound closure results for Mi‐2, Spt6, and Sin3A should be interpreted with caution pending development of additional reagents for verification of knockdown and/or localization.

To test if reporter expression coincides with the localization of a corresponding protein, we used a previously published (Srinivasan et al. 2008) commercially available anti‐Kismet antibody to stain wounded larvae (Fig. 2J−J′′′′). Kis reporter and Kis antibody staining overlapped and both showed diminished expression at the wound edge. However, Kis antibody staining was not significantly reduced by RNAi expression. Given the relatively weak knockdown achieved upon expression of *UAS‐Kis* ^*RNAi*^ (compare Fig. 2I with Fig. 2C,F) compared with other RNAi transgenes, we think it more plausible than not that the Kis reporter accurately tags Kis protein.

### Reporter lines tag proteins associated with both active and repressed chromatin

The trapped proteins we identified include factors that are thought to participate in both activation and repression of gene expression. Reporters for three proteins implicated in active gene transcription are downregulated during wound healing. Osa is a trxG protein and a member of the Brahma complex (Kennison and Tamkun 1988; Vazquez et al. 1999). A second trxG protein, Kismet, was also identified in our screen (Srinivasan et al. 2008). Spt6 is found at sites of active transcription; it functions in transcriptional elongation, and its localization on chromosomal sites is dependent on Kismet activity (Kaplan et al. 2000; Srinivasan et al. 2005). To test if clearance of the Spt6 reporter is also dependent on Kis, we expressed *UAS‐kis* ^*RNAi*^ in the epidermis (Fig. S3A−A′′′). Spt6 clearance from the wound edge was not abolished by expression of *UAS‐kis* ^*RNAi*^ (Fig. S3A′−A′′′) compared with control (Fig. S3A*).

Four reporters tagging proteins involved in repression of gene transcription were also cleared from wound edge cells during healing. All of these proteins exist in complexes containing or associated with histone deacetylase (HDAC) activity. Mip120 is part of the repressive *Drosophila* Rb, E2f and Myb (dREAM) complex which also associates with the HDAC Rpd3 (Beall et al. 2002; Lewis et al. 2004). Mi‐2 is an ATP‐dependent chromatin remodeling protein that participates in PcG‐mediated repression and interacts with Rpd3 (Kehle et al. 1998; Brehm et al. 2000; Khattak et al. 2002; Fasulo et al. 2012). Finally, Sin3A and Sap130 act together in a separate complex with HDAC activity supplied by Rpd3 (Pennetta and Pauli 1998; Spain et al. 2010). We tested if knockdown of Sap130 had any effect on Sin3A reporter clearance (Fig. S3B−C*) or, conversely, if expression of *UAS‐Sin3A* ^*RNAi*^ prevented Sap130 reporter clearance (Fig. S3D−D*). They were not interdependent (compare Fig. S3B′′′ to B*, S3C′′′ to C*, and S3D′′′ to D*). Interestingly, although Sap130 and Sin3A reporter clearance was not interdependent following wounding, we did find that their baseline levels of expression in unwounded epidermal tissue were slightly dependent upon each other (Fig. S3G,H).

### Temporal expression of epigenetic wound reporters

**Figure 3:**
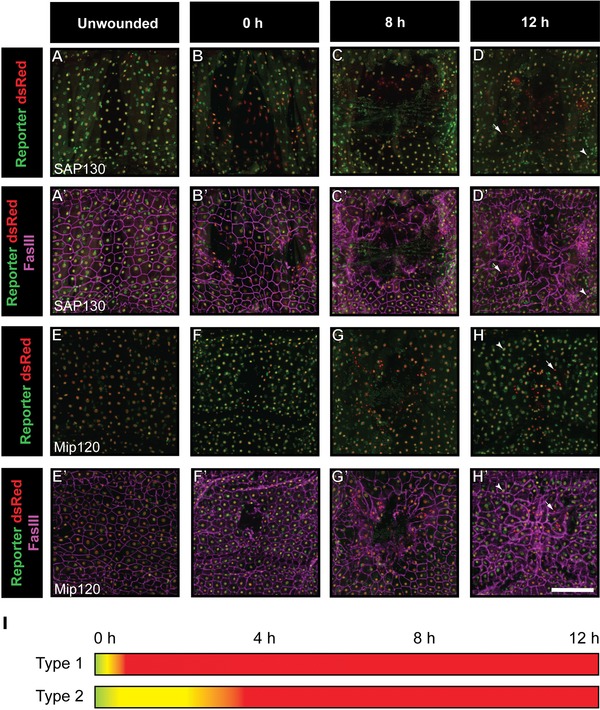
Two distinct temporal patterns of reporter regulation during wound healing. (A)−(H) Dissected epidermal whole mounts of larvae heterozygous for *e22c‐Gal4, UAS‐DsRed2‐Nuc* and the indicated reporter transgene were immunostained for Fasciclin III at the indicated times post‐wounding. Two examples are shown. In pattern 1 (A−D′, Sin3A^R2^ reporter), expression is lost from wound edge cells within minutes of wounding. In pattern 2 (E−H′, Osa^R1^ reporter), expression is lost between wounding and 4 h later. Scale bar 200 μm. (I) Graphical representation of reporter downregulation following wounding. Arrows indicate epidermal nuclei that have absent or reduced reporter expression. Yellow, epidermal nucleus expressing both DsRed and reporter; red, epidermal nucleus.

Next, we asked when the expression of each downregulated reporter first begins to diminish, and whether their expression is reestablished concomitant with wound closure. We therefore examined epidermal expression of each of the 13 reporters at 0, 8, and 12 h after wounding. We observed two types of expression pattern (Fig. 3I). Strikingly, the Sap130 and Sin3A reporters (type I) were cleared from wound edge cells within minutes of wounding (Figs. 2A−A′ and S3A−A′). Some nuclei that lack reporter expression can still be observed near the presumptive center of the closed wound 12 h later (arrows in Figs. 3D−D′ and S4D−D′). By contrast, reporters for Mip120, Kismet, Osa, Spt6, and Mi‐2 (type II) showed delayed clearance from wound edge cells, with the reporters present immediately post‐wounding (Figs. 3E−E′ and S4E−E′,I−I′,M−M′,Q−Q′) but absent at 4 h post‐wounding (Fig. 1A−D′′′,G−G′′′). However, as with type I reporters, weak or absent reporter staining was also observed at 12 h after wounding in most of these lines (Figs. 3H−H′ and S4H−H′,L−L′,P−P′,T−T′).

### Expression of epigenetic reporters is not controlled by known wound closure pathways

**Figure 4:**
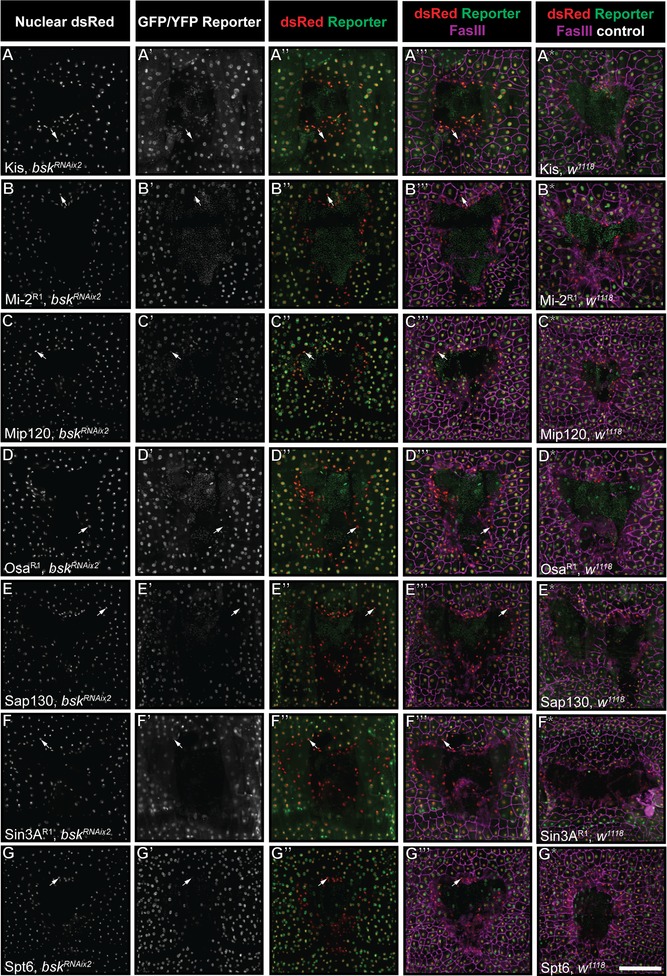
Wound reporter expression is not under the control of JNK. (A)−(G*) Dissected epidermal whole mounts of larvae heterozygous for *UAS‐DsRed2Nuc*, an epidermal *Gal4* driver, the indicated reporter transgene and either *bsk* ^*RNAix2*^ or *w* ^*1118*^ were immunostained for Fasciclin III at 4 h post‐wounding. One example for each protein is shown. Specific reporter lines and epidermal *Gal4* drivers are as indicated; see Table 1. Note that in each case diminished reporter expression can be observed at the wound edge (A′′−G′′ and A′′′−G′′′), similar to staining control when the *UAS‐bsk* ^*RNAix2*^ transgenes are absent (A*−G*). Arrows indicate examples of epidermal nuclei with absent or reduced reporter expression. Scale bar 200 μm.

**Figure 5:**
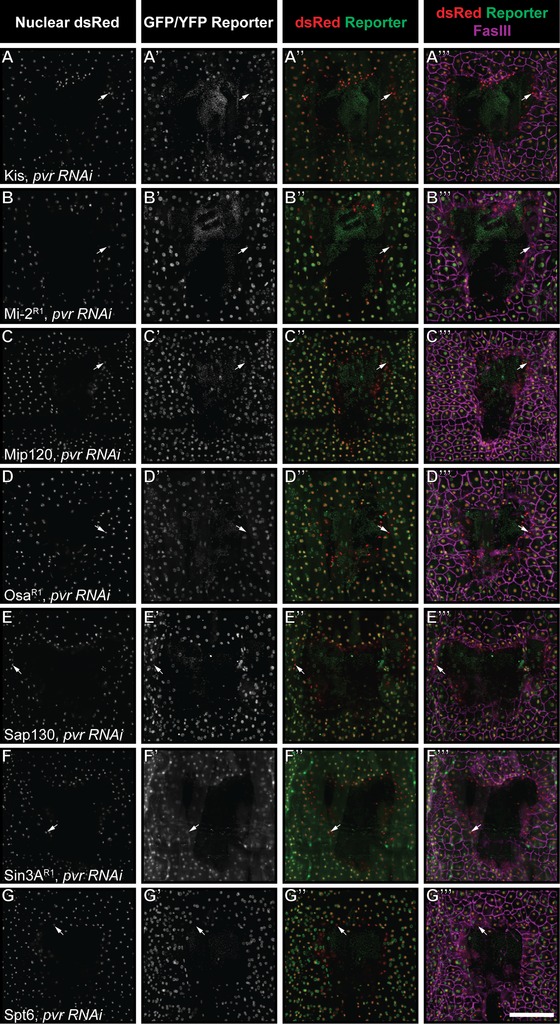
Wound reporter expression is not under the control of Pvr. (A)−(G′′′) Dissected epidermal whole mounts of larvae heterozygous for *UAS‐DsRed2Nuc*, an epidermal *Gal4* driver, the indicated reporter transgene and either *bsk* ^*RNAix2*^ or *w* ^*1118*^ were immunostained for Fasciclin III at 4 h post‐wounding. One example for each protein is shown. As in Figure 3, reporters on the third (Mi‐2, Osa, Sap130) and first (Spt6) chromosomes are combined with *e22c‐Gal4, UAS‐DsRed2nuc* while reporters on the second chromosome (Kis, Mip120, Sin3A) are combined with *A58‐Gal4, UAS‐DsRed2nuc*. Specific reporter lines are as indicated (see Table 1). Note that in each case diminished reporter expression can be observed at the wound edge (A′′−G′′ and A′′′−G′′′); compare with staining controls in Figure 3A*−G*. Scale bar 200 μm.

We asked whether diminished reporter expression is controlled by known wound healing pathways. Previous work by our laboratory and others has shown that the conserved JNK and Pvr pathways are required for wound closure in the fly larva (Galko and Krasnow 2004; Wu et al. 2009). We combined each epigenetic reporter line with one of two epidermal Gal4 lines (either *A58‐Gal4* or *e22c‐Gal4*; Galko and Krasnow 2004) driving nuclear DsRed (*UAS‐DsRed2Nuc*) to ensure the presence of the reporter and labeling of epidermal nuclei as in our previous experiments. We then crossed these animals to potent *UAS*‐*RNAi* lines targeting Pvr and JNK that had been shown previously to block wound closure (Wu et al. 2009; Lesch et al. 2010; Brock et al. 2012). This allowed us to assess, in progeny larvae bearing the appropriate transgenes, whether reporter expression is still diminished 4 h after wounding. For all seven tagged proteins, blocking JNK signaling via expression of *UAS‐bsk* ^*RNAi*^ had no obvious effect on loss of reporter expression in wound edge cells for any of the reporters examined (Fig. 4). This result was consistent across all additional reporter lines for Sin3A, Osa, and Mi‐2 (Fig. S5). Next, we blocked Pvr signaling via epidermal expression of *UAS‐Pvr* ^*RNAi*^. As with loss of JNK signaling, loss of Pvr signaling had no effect on wound edge reporter clearance in any of the 13 reporters examined (Figs. 5 and S6).

## Discussion

### Wiping the epigenetic slate clean to prepare for wound closure?

We describe 13 reporters trapping seven distinct proteins whose expression is rapidly diminished during wound closure. At the spatial level, the clearance of these reporters is largely restricted to wound edge cells. Taken together, these results suggest that larval wound closure requires decreased expression of two types of proteins: those associated with active chromatin, and those with repressive roles. It is possible that this reflects a necessity to shut down genes associated with the cells’ normal differentiated functions while turning on additional genes required for motility and other aspects of the wound response program.

What is perhaps more intriguing is that only downregulation of epigenetic reporters was observed. Why? Our previous work indicates that transcription of *msn*, *puc*, and *chic* is evident 4 h after wounding (Galko and Krasnow 2004; Brock et al. 2012). It is logical to suppose that upstream epigenetic changes affecting transcription would also be evident by this time. One possibility is that increased reporter expression is simply more difficult to detect than profound loss of reporter expression in our screening assay. It is also possible that reporters for upregulated proteins were not represented among the incomplete collection that we screened (which covered <18% of total epigenetic factors that could be tagged). For example, in the mammalian epidermis, both *Utx* and *Jmjd3* were upregulated in healing wounds (Shaw and Martin 2009a), but the sole *Drosophila* homolog of these proteins, *dUtx*, was not present in either reporter collection and therefore not screened here.

At the biological level, the active removal of select epigenetic proteins may be the most efficient way for the cell to quickly execute a dramatically changed transcriptional program. For many chromatin modifications, one protein exists to make the mark and a second protein exists to remove it. For example, histone demethylases remove marks made by histone methyltransferases, and histone deacetylases remove acetyl groups added by acetyltransferases. Thus, in theory, levels of a particular chromatin mark can be increased in two different ways: by increasing expression of the protein or complex that makes it, or by removing/inactivating the protein that removes it. Pinch wounds in *Drosophila* larvae close within 24 h. Perhaps removal of specific histone modifying proteins, which might immediately destroy their resulting activity, is the fastest way to erase the collection of histone marks currently constraining transcription, thus liberating the cell to deal with the “emergency” situation of the wound. Upregulation of chromatin modifying proteins—a process that would require both transcription and translation—may simply be too inefficient a process for cells already coping with damage. It will be interesting to see whether such a widespread clearance of epigenetic factors also applies in other contexts requiring a rapid and emergent cellular transcriptional response.

### Reporter expression is not dependent on known wound closure pathways

Temporal analysis of reporter expression revealed two distinct patterns of clearance from wound‐adjacent cells. Sap130 and Sin3A reporters were downregulated immediately after wounding, while expression of the remaining reporters diminished some time between the initial wounding and 4 h. It is interesting that Sin3A and Sap130 act together in the same HDAC‐containing complex (Spain et al. 2010). Such rapid clearance of these reporters from cells suggests an active protein degradation mechanism, rather than a diminished transcriptional activity that could be observed only after loss of both mRNA and proteins from the cells. Furthermore, these results suggested the possibility that the two temporal patterns may correspond to regulation by two different wound closure pathways. However, the independence of all reporter clearances from JNK and Pvr signaling raises the intriguing possibility that loss of reporter expression is under the control of one or more as‐yet‐undiscovered wound‐induced signals. Two possibilities for this early clearance signal are calcium and hydrogen peroxide which have been shown in multiple systems and assays to mediate early wound migratory responses (Niethammer et al. 2009; Moreira et al. 2010; Juarez et al. 2011; Xu and Chisholm 2011; Yoo et al. 2012; Enyedi et al. 2013; Razzell et al. 2013; reviewed by Wood 2012; Xu et al. 2012; Cordeiro and Jacinto 2013; Enyedi and Niethammer 2013).

### Implications for the epigenetic landscape during wound healing and regeneration

In this study, we have used reporters to gain insight into epigenetic regulation of wound closure. There are tradeoffs to this approach. Only a subset of the genes of interest have as yet been tagged, and furthermore it is possible that in some cases tagging alters protein function. However, our approach has clear benefits in terms of speed and economy and has allowed a much larger scale investigation of protein expression and localization than would be possible with antibody staining in this or other systems. In mice, the PcG proteins Eed, Ezh2, and Suz12 are downregulated during wound healing (Shaw and Martin 2009a). The *Drosophila* orthologs of these proteins were not represented in either the CPTI or Flytrap collections, and therefore it is not known if they are also downregulated in the fly during the larval wound response. However, some clues to their importance may be found in studies of regeneration. In muscle cells, Ezh2 represses Pax7 in response to inflammatory cues (Palacios et al. 2010). This repression of Ezh2 is deleterious to satellite cell proliferation. In zebrafish, the histone demethylase and Jmjd3 homolog kdm6B are required for fin regeneration (Stewart et al. 2009).

We found four reporters corresponding to proteins that interact, directly or indirectly, with the HDAC Rpd3: Sin3A, Sap130, Mip120, and Mi‐2. Could clearance of these proteins result in increased histone acetylation at wound responsive loci? An important role for histone deacetylases in regeneration has been noted by several groups but a general consensus on their role in regeneration is lacking. In the damaged kidney, for example, expression of HDACs inhibits *BMP7* expression and is deleterious to effective regeneration (Marumo et al. 2008). By contrast, HDAC activity is necessary for proliferation, but not for differentiation, of ear hair cells in the regenerating chick utricle (Slattery et al. 2009). HDAC activity is also necessary for limb and tail regeneration in frog and axolotl (Taylor and Beck 2012; Tseng and Levin 2012). An important difference between these regeneration models and larval epidermal wound healing is the lack of proliferation in the latter. It is conceivable that in this specific context HDAC activity is inhibitory to wound healing. In fact, with cell division out of the equation, this model may have the potential to tease out the specific roles of epigenetic regulation in cell migration and dedifferentiation.

### Utility of reporter screening

The Flytrap and CPTI collections constitute a unique public resource that tags a subset of *Drosophila* proteins. This resource can and should be used, as here, to survey classes of proteins of interest in any biological process amenable to visualization. With the growing density of Minos insertions (Bellen et al. 2011) in the fly genome that allow recombination‐mediated cassette exchange based tagging (Venken et al. 2011), this resource should expand to include many more proteins in the near future.

## Materials and Methods

### Fly stocks

*w^*1118*^;e22c‐Gal4, UAS‐DsRed2nuc20/CyO* (Lesch et al. 2010) and *w^*1118*^;UAS‐DsRed2nuc, A58‐Gal4/TM6B* were used to label the larval epidermis in dissected preparations. CPTI reporters maintained at the *Drosophila* Genetic Resource Center, Kyoto Institute of Technology, and used in this study were *w^*1118*^;PBac[602.P.SVS‐1]Mi‐2^*CPTI000020*^*, *w^*1118*^;PBac[681.P.FSVS‐1]Mi‐2^*CPTI001119*^*, *w^*1118*^;PBac[602.P.SVS‐1]Mi‐2^*CPTI000106*^*, *w^*1118*^;PBac[754.P.FSVS‐0}]osa^*CPTI003089*^*, *w^*1118*^;PBac[681.P.FSVS‐1]Sap130^*CPTI001478*^*, and *w^*1118*^;PBac[768.FSVS‐0]kis^*CPTI003576*^*, in addition to those listed in Table S1. Additional reporters from the Flytrap collection maintained in the laboratories of William Chia, Lynn Cooley, and Allan Spradling were *Mi‐2^*CA06598*^*, *Mi‐2^*YD0067*^*, *Sin3A^*P01869*^*, *Sin3A^*YB0058*^*, *Mip120^*P02006*^*, *Spt6^*CA07692*^*, and *osa^*CC00445*^*, as well as additional lines listed in Table S1. For simplicity, individual reporter lines will be referred to by the designations in Table 1 throughout this paper. *UAS‐pvr^*RNAi*^* and *UAS‐bsk^*RNAix2*^* (Lesch et al. 2010) were used to inhibit the JNK and Pvr pathways, respectively. The following lines were obtained from the Vienna *Drosophila* RNAi Center: *UAS‐kis^*RNAi*^* (*w^*1118*^;P[GD16331]v46685*); *UAS‐osa^*RNAi*^* (*w^*1118*^;P[GD1502]v7810*); *UAS‐Sin3A^*RNAi*^* (*w^*1118*^;P[GD4441]v37684/CyO*); *UAS‐Mi‐2^*RNAi*^* (*w^*1118*^;P[GD4511]v10766*); *UAS‐Mip120^*RNAi*^* (*w^*1118*^;P[GD11805]v35060*); *UAS‐Sap130^*RNAi*^* (*w^*1118*^;P[GD7168]v31394* and *w^*1118*^;P[GD7168]v31395*); *UAS‐Spt6^*RNAi*^* (*w^*1118*^;P[GD7536]v31701/TM3* and *w^*1118*^;P[GD7536]v31703/TM3*). *UAS‐osa^*RNAi*^* (*y^1^v^1^;P[TRiP.JF01207]attP2*) was obtained from the Transgenic RNAi Project collection maintained at the Bloomington *Drosophila* Stock Center.

### Wounding assays, dissection, and staining

*Drosophila* larvae were reared on standard cornmeal medium, anesthetized with ether, and wounded at mid‐third instar as described previously (Galko and Krasnow, 2004) except that for time‐course analysis smaller wounds than usual were made. All *Drosophila* were reared at 25°C unless otherwise noted. After wounding, larvae were allowed to recover on food for the indicated periods prior to dissection. Dissected larvae were fixed in 3.7% formaldehyde in phosphate‐buffered saline for 1 h, and then washed and stained as described previously (Burra et al. 2013). Antibodies used were mouse anti‐Fasciclin III, 1:100 (Developmental Studies Hybridoma Bank, Iowa City, IA, USA); rabbit anti‐GFP, 1:1000 (Life Technologies); goat anti‐Kismet, 1:200 (Santa Cruz, CA, USA). After dissection and staining, four fillets of each genotype were mounted in Vectashield (Vector Laboratories, Burlingame, CA, USA) prior to confocal microscopy. To determine the wound phenotype of homozygous larvae, three replicates of at least 10 larvae each were analyzed by live microscopy 24 h post‐wounding.

### Microscopy and image processing

Some images for the 4 h time‐point were obtained on an Olympus FV500 confocal microscope and saved in the Olympus MultiTIFF format. All remaining images were obtained on an Olympus FV1000 Confocal Microscope and saved in the Olympus OIB format. All images were captured using a 10× dry objective lens at 1.5× zoom using the optimal section size. To reduce signal from non‐epidermal tissues, only sections containing DsRed labeled nuclei, plus two to three sections on either side, were acquired. All images were processed in ImageJ using either the LOCI Tools plugin (for OIB format) or the UCSD Tools plugin (for MultiTIFFs). Each channel was stacked separately with Sum Slices, converted to RGB mode, and saved as TIFFs.

TIFFs were imported into Adobe Photoshop as screen‐mode layers. All manipulations were applied globally across the entire image. A duplicate was made of each layer and colorized to red, green, or magenta. Because FasIII staining varies considerably across the wound epithelium, with highly intense staining at the wound edge that can obscure signal from other channels, a modification of a previously published algorithm (Sedgewick 2008), was used to equalize the FasIII signal. Briefly, the magenta FasIII layer was copied. Gaussian Blur was applied to the new layer and the layer was inverted. The layer mode was changed to “Soft Light” and merged with the original magenta FasIII layer. Use of this algorithm is purely for visualization of wound and cell morphology, and no comparisons of FasIII levels should be made within or between images. For preparation of figures, brightness and contrast were adjusted where necessary.

### Quantification of reporter expression

Combinations of each reporter with *UAS‐RNAi* lines and *w* ^*1118*^ controls were dissected on the same day and combined into a single tube after fixation to ensure identical staining conditions. For all measurements of reporter intensity, raw *Z*‐projections of dsRed and GFP channels were imported into ImageJ. For each reporter line, the threshold was set identically on each dsRed image to identify epidermal nuclei. Measurements were set to find the *X* and *Y* coordinates of each particle, as well as its area and mean gray value, redirected to the GFP image. Analyze Particles was used to retrieve all measurements from particles of size 20–200 μm. An average nuclear intensity was calculated for each image. The mean intensity was then calculated from three to four images for each genotype. Knockdown images were compared with controls using Student's *t* test.

## Supporting information

Additional Supporting Information may be found in the online version of this article at the publisher's website:

**Figure S1**. The Dp reporter lacks wound‐specific expression. (A)−(D) Dissected epidermal whole mounts of larvae heterozygous for *e22c‐Gal4, UAS‐dsRed2nuc* and Dp reporter CA06594 4 h after wounding. Wound edge nuclei that fail to express the Dp reporter were not observed (A, B, C). Arrows, wound edge nuclei expressing both dsRed and GFP. Scale bar 200 μm.

**Figure S2**. Additional reporters downregulated in the vicinity of the wound. (A)−(F′′′′) Dissected epidermal whole mounts of larvae heterozygous for *e22c‐Gal4, UAS‐DsRed2‐Nuc* and the indicated reporter transgene were immunostained for Fasciclin III 4 h post‐wounding. As in Figure 1, for all genotypes, DsRed panels (A−F), (A′′−F′′), (A′′′−F′′′) show the location of epidermal nuclei while reporter panels (A′−F′), (A′′−F′′), (A′′′−F′′′) highlight all cells expressing the reporter. Fasciclin III staining (magenta, A′′′−F′′′) highlights epidermal cell membranes and the wound edge. All animals are heterozygous for *e22c‐Gal4, UAS‐DsRed2nuc* and the reporter lines indicated (see Table 1). Arrows indicate epidermal nuclei that have lost detectable reporter expression. Scale bar 200 μm.

**Figure S3**. Reporters in the same complex are not interdependent for wound edge clearance. (A)−(F*) Dissected epidermal whole mounts of larvae heterozygous for *A58‐Gal4* or *e22c‐Gal4, UAS‐DsRed2Nuc* and the indicated reporters and RNAi lines were immunostained for Fasciclin III (magenta) and GFP (green). Spt6 and Sin3A larvae were raised at 30°C prior to wounding to maximize RNAi expression, while Sap130 larvae were raised at 25°C to maximize viability. Regardless of rearing temperature, all larvae were allowed to recover from wounding for 4 h at 25°C. (A)−(A*) Knockdown of Kis does not prevent Spt6 reporter clearance. (B)−(B*), (C)−(C′′′), (D)−(D*), (E)−(E′′′) Knockdown of Sap130 does not prevent Sin3A reporter clearance. (F)−(F*) Knockdown of Sin3A does not prevent Sap130 reporter clearance. Arrows in (A)−(F*) indicate wound‐proximal epidermal nuclei that have lost reporter expression. Scale bar 200 μm. (G), (H) Quantification of reporter intensity in unwounded epidermal segments from larvae heterozygous for *A58‐Gal4* or *e22c‐Gal4, UAS‐DsRed2Nuc* and the indicated reporters and *UAS‐RNAi* lines. Sin3A reporter larvae were raised at 30°C, while Sap130 reporter larvae were raised at 25°C, as in (A)−(F*) above. (G) Intensity of Sin3A^R1^ reporter with epidermal expression of the indicated *UAS‐Sap130* ^*RNAi*^ lines. (H) Intensity of Sap130 reporter with expression of *UAS‐Sin3A* ^*RNAi*^. Asterisks indicate *P* < 0.05.

**Figure S4**. Temporal patterns of additional proteins during wound healing. (A)−(L′) Dissected epidermal whole mounts of larvae heterozygous for *e22c‐Gal4, UAS‐DsRed2‐Nuc* (red) and the indicated reporter transgene were immunostained for Fasciclin III (magenta) at the indicated times post‐wounding. Temporal expression for one example of each of the proteins absent from Figure 2 is shown; time‐points are as indicated except for Kismet, where due to delayed closure the 12‐h time‐point is shown in (K) and (K′) and the 24‐h time‐point in (L) and (L′). Arrows indicate epidermal nuclei near the presumptive center of the wound that lack reporter expression; arrowheads indicate epidermal nuclei distal to the wound with robust reporter expression. Genotypes of all animals are *e22c‐Gal4, UAS‐dsRed2nuc/*+ and include the reporter lines indicated (see Table 1). Scale bar 200 μm.

**Figure S5**. Additional wound reporters are not under the control of JNK. (A)−(F*) Dissected epidermal whole mounts of larvae heterozygous for *UAS‐DsRed2Nuc*, an epidermal *Gal4* driver, the indicated reporter transgene and either *bsk^RNAix2^* or *w* ^*1118*^ were immunostained for Fasciclin III at 4 h post‐wounding. One example for each protein is shown. Specific reporter lines and epidermal *Gal4* drivers are as indicated; see Table 1. As in Figure 3, diminished reporter expression can be observed at the wound edge (A′′−F′′ and A′′′−F′′′), similar to control (A*−F*). Arrows indicate examples of epidermal nuclei with absent or reduced reporter expression. Scale bar 200 μm.

**Figure S6**. Additional wound reporters are not under the control of Pvr. (A)−(H*) Dissected epidermal whole mounts of larvae heterozygous for *UAS‐DsRed2Nuc*, an epidermal *Gal4* driver, and indicated wound reporters not shown in Figure 4. Specific reporter lines are as indicated (see Table 1). Note that in each case diminished reporter expression can be observed at the wound edge (A′′−H′′ and A′′′−H′′′); compare with Figure S3A*−GH*. Scale bar 200 μm.

**Table S1**. Additional reporter lines tested in the screen.

**Graphical Table**
